# Perception of providers on use of the WHO mental health Gap Action Programme-Intervention Guide (mhGAP-IG) electronic version and smartphone-based clinical guidance in Nigerian primary care settings

**DOI:** 10.1186/s12875-022-01869-7

**Published:** 2022-10-15

**Authors:** Akin Ojagbemi, Stephanie Daley, Lola Kola, Tatiana Taylor Salisbury, Yvonne Feeney, Akerke Makhmud, Heidi Lempp, Graham Thornicroft, Oye Gureje

**Affiliations:** 1 World Health Organization Collaborating Centre For Research And Training In Mental Health, Neuroscience, And Substance Abuse, Department of Psychiatry, College of Medicine, University of Ibadan, Ibadan, Nigeria; 2 Centre for Dementia Studies, Brighton and Sussex Medical School, Brighton, UK; 3 Centre for Global Mental Health, Health Service and Population Research Department, Institute of Psychology, Psychiatry and Neuroscience, King’s College London, London, UK; 4 Centre for Rheumatic Diseases, Department of Inflammation Biology, School of Immunology and Microbial Sciences, Faculty of Life Sciences and Medicine, King’s College London, London, UK

**Keywords:** Psychosocial interventions, Remote supervision, Sub-Saharan Africa, Task sharing

## Abstract

**Background:**

Taking advantage of the rapidly increasing access to digital technology in low- and middle-income countries, the World Health Organization has launched an electronic version of the mental health Gap Action Programme intervention guide (emhGAP-IG). This is suitable for use on smartphones or tablets by non-specialist primary healthcare providers (PHCWs) to deliver evidence-based intervention for priority mental, neurological and substance use disorders. We assessed the perceptions of PHCWs on the feasibility, acceptability, and benefits of using smartphone-based clinical guidance and the emhGAP-IG in the management of people with mental health conditions in Nigeria.

**Methods:**

Exploration of the views of PHCWs from 12 rural and urban primary health clinics (PHCs) in South-Western Nigeria were carried out using 34 in-depth key informant qualitative interviews with nurses (*n* = 10), community health officers (*n* = 13) and community health extension workers (*n* = 11). An additional two focus group discussions, each comprising eight participants drawn from across the range of characteristics of PHCWs, were also conducted. Thematic analysis was conducted using a three-staged constant comparison technique to refine and categorise the data.

**Results:**

Three overall themes were identified around the use of clinical guidance and mobile applications (apps) in PHCs. Apps were deployed for purposes other than clinical consultation and decision making. Although paper-based guidance was the expected practice, its utilization is not fully embedded in routine care. An app-based decision-making tool was preferred to paper by PHCWs. Future usage of the emhGAP-IG would be facilitated by training and supporting of staff, helpful design features, and obtaining patients’ buy-in.

**Conclusion:**

Our findings suggest that the emhGAP-IG could be a viable way to embed clinical guidance and decision-making tools in the management of people with mental health conditions in Nigerian PHCs.

**Supplementary Information:**

The online version contains supplementary material available at 10.1186/s12875-022-01869-7.

## Introduction

Globally, approximately 90% of people with untreated mental health conditions live in low- and middle-income countries (LMICs) [1] where 80% of all people with a mental health disorder also reside [2]. Evidence from the World Mental Health Surveys [3, 4] suggested that in Nigeria, only 0.8% of persons with anxiety disorders and 6% of those with mood disorders made any treatment contact in the year of the illness. The majority of Nigerians who sought treatment did so at primary or general healthcare settings [5]. Scarcity of mental health specialists and the concentration of hospitals with mental health capabilities in geographically distant urban locations are two factors that rank as top barriers to accessing specialist mental health care in LMICs [6].

‘Task sharing’ [7] is one of the efficient solutions to the challenge of providing adequate care to people with mental health disorders in LMICs. This involves strengthening the capacity of non-specialist healthcare workers to deliver evidence-based mental healthcare with support and supervision from better trained or specialist care providers [7]. Non-specialist healthcare workers have the advantage of being more widely available. They often live in the same community as patients and share patients’ explanatory models of illness [8]. They are thus better able to deliver basic psychosocial interventions using culturally appropriate approaches that are more acceptable to patients. Several randomised controlled trials (RCT), reviewed by Singla and colleagues [9], have demonstrated the effectiveness of psychosocial interventions delivered by non-specialist healthcare workers for mental health conditions in LMICs [10].

Task sharing require the availability of evidence-based tools. Based on over 90 systematic reviews [11, 12], the Mental Health Gap Action Programme-Intervention Guide (mhGAP-IG) v1.0 was launched by the World Health Organization (WHO) as a clinical support instrument to assist non-specialist mental health providers in primary and community health services located in LMICs to deliver evidence-based interventions for priority mental, neurological and substance use disorders [12]. Since its launch in 2010, the mhGAP-IG v1.0 has been used in over 100 countries worldwide. It has been found to be effective for the delivery of evidence-based mental health services within primary healthcare in LMICs [10].

Following a consultation exercise to assess the 5-year impact of the mhGAP-IG, the WHO developed and launched the e-version of the mhGAP-IG (v2.0) in October 2017, as an application (App) suitable for both iOS and Android smartphones and tablets. The mhGAP-IG v2.0 (e-mhGAP-IG) consists of 8 modules addressing priority conditions including depression, psychosis, epilepsy, child and adolescent mental and behavioural disorders, dementia, disorders due to substance use, self-harm or suicide, and other significant mental health complaints that impair daily functioning or lead to help seeking). Each module includes a summary of the common presentations of the relevant condition as well as guidance for assessment, management and follow-up. One of the advantages of the e-version is an enhanced opportunity for quality improvement through remote supervision. However, there is limited literature on its use to date. The most recent updated systematic review of implementation of the WHO mhGAP-IG and its associated tools found one trial registration for the comparison of the e-version with the paper mhGAP-IG [10].

There is a rapidly increasing access to mobile technology and smartphone-based applications in many LMICs. The Groupe Speciale Mobile Association [13] suggest that between 2012 and the end of 2020, the number of unique mobile phone subscriptions in sub-Saharan Africa (SSA) increased by over 42%. Approximately 46% of the region’s population currently subscribe to mobile technology. This proportion is predicted rise to 50% by 2025, at which time 70% of total connection in West Africa will be via a smartphone. Mobile penetration in Nigeria was projected to increase to 55% in 2025 [14]. Thirty-six percent of mobile phone connections in Nigeria are currently linked to a smartphone. Mobile technology provides opportunities to expand capacity of the few available mental health specialists in SSA to offer training, support and supervision of nonspecialist healthcare workers who are providing mental health services to communities, especially those that are distant from the urban centres where specialized facilities are commonly based [15].

The mhGAP-IG was adopted, in 2013, by Nigeria’s highest health policy making body as a pathway for scaling up mental health services in the country [16]. Studies in Nigeria have demonstrated the use of mobile technology to support the delivery of evidence-based mental health interventions through task sharing with non-specialist health workers. For example, in the Expanding Care for Perinatal Women with Depression (EXPONATE) project [17], community midwives trained in parenting skills as well as in the basic treatment recommendations of the mhGAP-IG were supported by mental health specialists through mobile phone platforms to deliver weekly interventions to pregnant women who screened positive for depression. Follow-up assessments at six months post intervention showed remission rates of 66% to 70% depending on the intensity of intervention [17].

To further develop the e-mhGAP-IG for successful dissemination in primary healthcare settings, it is essential to understand the needs, contexts, and constraints of frontline primary healthcare workers (PHCWs) about current and future use of app-based clinical guidance. There are no previous studies assessing the feasibility of an app-based decision-making tool for mental health diagnoses in Nigerian primary care settings. The current study was conducted as a part of the UN Medical Research Council (MRC) funded **E**-**m**hGAP **I**ntervention guide in **L**ow and m**i**ddle-income countries: proof-of-concept for Impact and **A**cceptability (Emilia Project). The aim of the current sub-study was to explore the perceptions of mhGAP-IG trained Primary Health Care Workers (PHCWs) on the feasibility, acceptability, and benefits of using smartphone app-based clinical guidance and the emhGAP-IG for detection and treatment of mental health conditions in patients attending primary care.

## Methods

### Study design

We used a qualitative study design. This methods allowed for detailed exploration of personal views beyond what might be possible to elicit in a quantitative survey [18]. These, in the present study included details about the current practice in regarding the use of clinical guidance and apps, interest in or opposition to the emhGAP-IG and apps, facilitators and barriers to future deployment of app-based decision-making tools in primary healthcare. This information was gathered using Key Informant Interviews (KII) and Focus Group Discussions (FGD).

### Participants and settings

The study took place in 12 primary health clinics (PHCs) representing a range of urban and rural settings within four local government areas (L.G.As) in Ibadan metropolis, Southwestern Nigeria (Table 1). In all, Ibadan metropolis has 11 local government areas and a population of approximately 3.5 million people. The four LGAs included in the present study are Ibadan North, Ibadan North West, Lagelu and Akinyele.

**Table 1:** Profile of Primary healthcare clinics included in the study

**Clinics**	P**atient flow per month**	**Rural/urban distribution**
Aba Emu	1490	Rural
Alakia	1298	Rural
Ojoo	1200	Rural
Moniya	800	Rural
Odo-Ona Elewe	623	Rural
Oranyan	520	Urban
Apete	500	Urban
Alegongo	500	Rural
Oniyanrin	500	Urban
Apogbon	450	Urban
Oke Adu	350	Urban
Sango	350	Urban

Specialist mental health services in Ibadan are primarily provided by two large general hospitals. There are 186 PHCs each serving a population of approximately 10,000. PHCs in Ibadan and Nigeria are staffed by non-physician health workers: nurses, Community Health Officers (CHOs) and community health extension workers CHEWs). In Nigeria, CHOs and CHEWs receive 2–3 years post-secondary school training in basic health issues. They often have limited training in mental health. Non-physician PHCWs in Nigeria are authorized to prescribe a limited range of mental health medications, including amitriptyline and chlorpromazine. They are routinely supervised by either general physicians or senior nurses, and all are employed in the public health system.

### Study procedure

Participants were purposively selected staff of the 12 PHCs who had received training in the use mhGAP-IG and who provide treatment for common health conditions, including mental health disorders, presenting in primary care. The mhGAP-IG training workshop ran for five days. It was competency based with didactic lectures, role plays, pre-and post-training assessments. The main goal of training was on the use of mhGAP-IG in routine practice.

All participants in the present study provided informed and written consent to participate. We first conducted two FGDs, each comprising eight informants drawn from across the range of characteristics of PHCWs. This was to explore organizational, contextual and process issues. We next conducted thirty-four semi-structured KIIs with nurses (*n* = 10), community health officers (*n* = 13) and community health extension workers (*n* = 11). This was to explore personal experiences, including sensitive issues that respondents might have difficulty discussing at FGDs.

### Interview questions

We developed a contextually relevant interview topic guide for both the FGDs and KIIs. This was reviewed by a group of 3 PHCWs comprising a nurse, CHO and CHEW. The interview guide explored a range of topics such as daily work life, identifying key problems at work, use of technology at work, as well as use of and interest in supervision and support. The FGDs and KIIs were conducted between April and August 2019, in Yoruba and English languages, at the PHCs and by trained research assistants under the supervision of LK and AO. While the KIIs lasted between 45 and 60 min, the FGDs lasted for an hour and half. All sessions were audio-recorded with the permission of participants.

### Qualitative data analyses

Recordings of FGDs and KIIs were transcribed verbatim by research staff and anonymised to maintain confidentiality. Translation into English was caried out by two independent forward translators fluent in English and Yoruba languages. They compared their versions to identify discrepancies, use of ambiguous or vague wording and accuracy. A final version of the transcript in English was developed with the two original translators by a research staff fluent in English and Yoruba languages. Back translation to the source language was conducted by a fourth independent translator who was blinded to the original transcript.

**Fig. 1:**
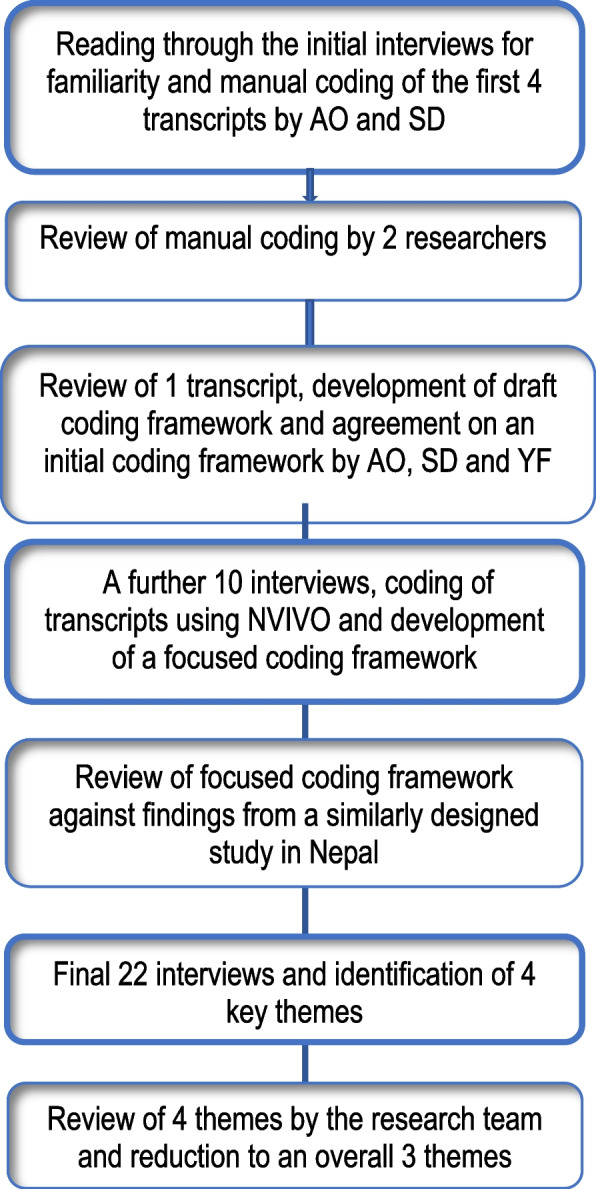
Process of qualitative data analyses

All final transcripts were analysed in three stages as summarised in Fig. 1 using thematic analysis [19]. Analyses started with two researchers (AO and SD) independently reading and re-reading transcripts for familiarity and to begin to identify pertinent themes. Each researcher labelled meaningful segment of text manually using descriptive codes. The first four KII transcripts were independently coded. The researchers met after each transcript was coded to review their respective preliminary codes to identify areas of similarities and differences. Disagreements in assignment of attribute codes was resolved through discussion to achieve consensus. A draft initial coding framework was then developed, reviewed, and agreed upon.

In the second phase, a further 10 transcripts were coded using the initial coding framework. Systematic collation and review of data within each code was enabled by the computer software package, NVivo 10 (QSR International, 2012). The qualitative data set was reviewed during frequent meetings between the researchers during which respective codes within new transcripts were compared with previous transcripts for rigor (Saldaña, 2009). A focussed coding framework was then developed in the third phase. These narrower codes were reviewed against findings from a similarly designed study conducted in Nepal [15]. Relationships between codes were also identified at this stage. The final phase of the analysis included 22 final interviews, from which four key themes were identified. These were reduced to three overall themes after review by the research team. Rigour in the analyses process was supported by frequent meetings between AO, SD and a third researcher (YF) to review coding, emergence of themes and reflexivity.

## Results

The characteristics of KII participants is presented in Table 2. Reflecting the distribution of PHCW and PHCs in Ibadan, the majority of KII participants were female and were drawn from urban PHCs (Table 2). Odo-ona and Ojo PHCs had the highest number of participants (*N* = 7 each), while Apogbon and Sango PHCs had the least representation (*N* = 1 each). In all, PHCWs in the FGDs comprised of six nurses, five CHOs and five CHEWs. Their ages ranged from 46–59 years and they had an average of 23 years of total work experience and 10 years of work with patients living with mental health conditions.

**Table 2:** Profile of primary health care workers selected for qualitative interviews in the study

**CHARACTERISTICS**	**AVERAGE**	**RANGE**
*Age*	46 years	36–55 years
*Years of work experience*	20 years	10–30 years
*Years of work in mental health*	4 years	1 -6 years
	**NUMBER**	**PERCENT**
*Sex*		
Female	29	85.3
Male	5	14.7
*Designation*		
Nurse	10	29.4
Community health officers	13	38.2
Community health extension workers	11	32.4
*Location of practice*		
Rural	15	44.1
Urban	19	55.9

### Key themes

The analyses of the interviews and FGDs generated three main themes around the use of clinical guidelines and apps in primary care: Current practice, Future practice, and Design suggestions (Table 3).

**Table 3:** Summary of themes from the Perception of providers about use of the mental health gap action program intervention guide and Apps in Nigerian primary care settings

**Theme**s	**Facilitator**s	**Barrier**s	**Some illustrative quotes**
Current Practice	• Apps used for purposes other than clinical consultation and decision making • Expectations of staff–*mandate, need to be accessible and use guidance*	• Workload • Systematic exclusion of some staff- *Low cadre, older, have no smartphone* • Patients’ expectation of staff to not use phone in consultation • Paper record of clinical activities • Paper guidance/mhGAP-IG not embedded	*‘I was the focal person for my LGA (Local Government area). App was used to send reports on immunization which involved the number of children immunized and the number of vaccines used…’*(Interview, Female, Nurse) *‘Mostly we don’t have time to use it during work hours; that is the fact.’*(Focus group participant, Female, CHEW) *….’though Community Health Extension Workers were excluded from the project.’*(Interview, Female, Nurse) *‘The only thing that Patients don’t like is for you to be making calls during consultations. They don’t like it at all.’*(Focus group participant, Male, CHO) *‘…. like the mh-GAP book given to us, I cannot remember the last time I opened it….’*(Interview, Female, Nurse)
Future Practice	• Smartphone guidance preferred to paper- *Sees many benefits* • Training • Patient’s expectation of staff to explain process	• Embedded practice- *E.g., Staff exclusion as above, interest*	*‘To me, putting it (mhGAP-IG) on the electronic (platforms) is better in the sense that it will make it easy for people to go through it (e-mhGAP-IG) at any time of the day’*(Interview, Female, Nurse) *‘The only thing that can solve that problem is there will be training, there must be a training on that aspect before applying it…’*(Interview, Female, Nurse) *‘You know, before you start with the patient, you can explain to them that once they see you doing things on the phone, they should not think you are not listening to them. It is actually for their good. You tell them that answers to some of the questions they ask, may be found on the device on your hand as the health worker.’*(Interview, Female, CHO)
Design features	• Features to encourage utility		*‘Supervision too can be there when there is a checklist there. And anytime you want to supervise, that one will give you the clue of what and what you are looking for like the time we were asked to go for the supervision after the training of SPECTRA (Another programme)’*(Focus group participant, Female, Nurse) *‘Video of somebody depressed but has overcome it [depression] can be of help, by letting them [service users] see it [recovery story] that they [service users] can come out of that problem [depression] if they [service users] cooperate with us [providers], and also true life experience can help, that will serve as client expert interview…’*(Interview, Female, Nurse) *‘….just like the NURI (another programme)App, the checklist [of signs and symptoms] should be there…’*(Focus group participant, Female, Nurse) *‘Like all those core signs can be there (in the App), the other signs can be there (in the App) and even the managements can be there too and if possible…’*(Interview, Female, Nurse)

*MhGAP-IG* Mental health gap action programme intervention guide, *CHEW* Community health extension worker, *CHO* Community health officer

#### Theme 1: Current practice

This theme related to current practice of using clinical guidelines and apps in primary care and included both facilitators and barriers influencing their use. We will next consider the sub-themes.

##### Facilitators of current use of guidelines and apps in primary care

Participants described how apps were used in practice. They reported that apps were deployed for purposes other than clinical consultation and follow-up, in particular, for making and receiving phone calls, official communication, collection and transmission of clinical data for surveillance.

‘I was the focal person for my LGA (Local Government Area). (The) app was used to send reports on immunization which involved the number of children immunized and the number of vaccines used…’ (Interview, Female, Nurse).

Several facilitators of current use of apps and clinical guidelines were identified in the interviews and focus group discussions. These included formal directives to own a smartphone, to keep the devices powered and to not run out of data, expectations for staff to be accessible via their mobile devices, and staff anticipation of official communication at any time of the day. Consequences of non-compliance with these requirements included disciplinary phone calls from managers. Staff reported that there was substantial compliance with official mandates. Compliance with official directives about use of designated smartphone apps appeared to have supported a change of practice.

‘During the programme, they [programme officials] mandated (it for) every health worker involved to get a working android phone, and that they [health workers] must not run out of data because messages can pop up any time, even midnights. It was also mandated for them [health workers] to get a working power bank to keep powering their phones always.’ (Interview, Male, CHO).

Staff reported that it was part of their expectation to have and consult clinical guidance and protocols as part of their work. One such protocol is the ‘Standing Order’ paper guidance for general clinical practice in Nigerian primary care settings. The document describes the scope of services offered in primary care and provides an overall guidance for the work of primary healthcare workers. Participants reported that some staff also used app-based reference guides for purposes such as antenatal and post-natal care as well as immunization services within primary care.

‘I use it [the app] to send data [to the central collation office]; last year I remember one case……, bleeding was much. I learnt from there (App) about the type of treatment I should give when matters happen like that.’ (Focus group participant, Female, CHEW).

##### Barriers to current use of paper guidance and apps in primary care

Participants reported that busy workloads were a barrier to using existing paper guidance such as the ‘Standing Order’ and mhGAP-IG v1.0 within current practice. They also suggested that patient expectations about phone use was a reason for not using their phones during consultations. It was reported that patients disliked telephone calls during their own consultations, which could potentially limit the use of app-based decision-making tools and emhGAP-IG during consultations.

‘The only thing that patients don’t like is for you to be making calls during consultations. They don’t like it at all.’ (Focus group participant, Male, CHO).

Participants described the practice of excluding certain groups of staff from the requirement to use designated apps. This group of staff included those who did not own a smartphone or were unable to operate one, older staff and those belonging to a lower cadre (CHEWS).

….’though Community Health Extension Workers were excluded from the project.’ (Interview, Female, Nurse).

Participants reported that notes from patients’ consultation appointments were currently kept in paper form. They also expressed that use of the paper version of the mhGAP-IG v1.0 was not routinely embedded into current clinical practice. This was despite a perception that it was easy to deploy due to its diagnostic and treatment algorithms. Reasons given for its low utility were time and work conflicts, as well as a perception that it may appear unprofessional to consult a paper guidance during patients’ consultation.

‘Mostly we don’t have time to use it (Apps and Guidelines) during work hours; that is the fact.’ (Focus group participant, Female, CHEW).

#### Theme 2: Future practice

This theme related to potential facilitators and barriers to future deployment of the app.

##### Facilitators of future use of guidelines and apps in primary care

Several participants suggested that future utilization of paper guidelines including the mhGAP-IG v1.0 may be facilitated by incorporating helpful algorithms that could be visibly positioned in consulting areas. The potential for the mhGAP-IG to be accessed via smartphone or tablet (e-mhGAP-IG) was seen as positive and preferred to the existing paper version. Many staff reported having smartphones which they carry around and frequently use. Participants identified several benefits of an e-mhGAP-IG: including better accessibility, ease of use, potential to incorporate support and supervision, and the device (smartphone or tablet) being more prestigious as a clinical practice tool. They perceived that the emhGAP-IG will be more acceptable to patients.

‘To me, putting it [the mhGAP-IG] on the electronics is better in the sense that it will make it easy for people to go through it [the mhGAP-IG] at any time of the day’ (Interview, Female, Nurse).

##### Barriers to future use of guidelines and apps in primary care

Barriers identified previously such as not all staff owning smart phones, particularly older staff and workload were also seen as barriers to future use of guidance and apps. Additionally, reluctance to adopt new ways of working, and a desire for consistency were identified.

‘…I feel that at times, the phone may be faulty. I will prefer to use paper because if the phone should be faulty, I can still be able to refer to it [the mhGAP-IG] on paper. It [the paper version of the mhGAP-IG] is easy to refer to, and then paper is more constant than phone….’ (Interview, Female CHO).

##### Facilitators of a future app-based mhGAP-IG

Several focus group and interview participants reported the need for improved support and supervision for their clinical work. Current supervision and support are typically unstructured and provided by a supervisory general practitioner to a group of 6–8 clinics in a local government area. Participants reported that there was low availability of supervisors as well as limited supervisory sessions due to the time required by supervisory physicians to travel to conduct such sessions. Participants reported enthusiasm about the potential for a smartphone mhGAP-IG in facilitating support and supervision.

‘Supervision too can be there when there is a checklist there. And anytime you want to supervise, that one [the supervision interphase] will give you the clue of what and what you are looking for like the time we were asked to go for the supervision after the training of SPECTRA (another health programme).’ (Focus group participant, Female, Nurse).

Several of the interview participants reported that use of e-mhGAP-IG would be facilitated by training of staff. There was a suggestion that training should involve all staff regardless of their organisational level. Participants stressed that CHEWs should be not excluded, as had happened previously, and highlighted the value of their inclusion due to their regular smart phone use. Participants also reported about a positive previous experience of training all cadre of staff in an antenatal care programme. Participants also linked staff trainability and use of an app-based mhGAP-IG to a younger age of the staff as well as interest in using smartphones.

‘The only thing [factor] that can solve that problem [of lack of use of the emhGAP-IG] is there will be training, there must be a training on that aspect [the app] before applying it [the emhGAP-IG]…’ (Interview, Female, Nurse).

Several participants reported that the emhGAP-IG should be introduced to patients both during a health education session conducted before actual clinical consultation and during routine consultations. The need to explain the process, and benefits of using the app to patients was stressed. Explaining the process of use of the e-mhGAP-IG was perceived to be important to generate patient acceptability of the tool as well as confidence in the skills of the healthcare worker and their ability to make correct decisions about their health conditions. It was felt that this would provide assurance that use of the e-mhGAP-IG during consultation would be to the patients’ overall benefit.

‘You know, before you start with the patient, you can explain to them that once they [service users] see you [the provider] doing things on the phone, they [service users] should not think you [provider] are not listening to them [service users]. It is actually for their [ervice users] good. You [the provider] tell them [service users] that answers to some of the questions they [service users] ask, may be found on the device on your hand as the health worker.’ (Interview, Female, CHO).

#### Theme 3: Design suggestions

This theme was not associated with sub-themes.

Participants suggested several design features that would facilitate use of an e-mhGAP-IG. The need for easy navigability was suggested, as was the inclusion of a reference guide, training videos, patient recovery stories, a checklist of signs and symptoms, a client follow-up system as well as support and supervision systems.

‘Video of somebody depressed but has overcome it [depression] can be of help, by letting them [service users] see it [recovery story] that they [service users] can come out of that problem [depression] if they [service users] cooperate with us [providers], and also true life experience can help, that will serve as client expert interview…’(Interview, Female, Nurse).

‘….just like the NURI (another programme) app, the checklist [of signs and symptoms] should be there [the emhGAP-IG app]…’(Focus group participant, Female, Nurse).

## Discussion

We have found in the present study that smartphone-based apps were used in Nigerian primary care settings, but for purposes other than clinical consultation and decision making. Use of designated apps in these settings was facilitated by official directives, which were complied with by most staff. However, it was suggested that older staff (i.e., closer to retirement), those who were unable operate a smartphone, and those belonging to a lower organisation cadre (CHEWs) were often exempt from official directives to use designated smartphone-based apps. Even though it was part of patients and staff expectation to use clinical guidelines for their work, the mhGAP-IG v1.0 (paper mhGAP-IG) and other existing paper guidelines were not fully embedded in current practice. A smartphone based mhGAP-IG was preferred to the paper format and it was perceived that future use would be facilitated by training and re-training of staff, helpful design features, and obtaining patients support by explaining the process, as well as the benefits during clinical contact.

Our findings are in keeping with previous reports from other LMICs. For example, a similarly designed study conducted as part of the EMILIA project in central Nepal [15] reported that PHCW demonstrated high levels of interest in a mobile app-based clinical guidance (mhGAP-IG v2.0) to support the identification and treatment of mental health disorders. Apart from convenience and ease of use, primary health care workers in Nepal also saw the possibility for remote supervision as a potential benefit of the mhGAP-IG v2.0 [15]. Important differences in result of the two studies is that while PHCW in Nigeria and Nepal expressed enthusiasm with the e-version of mhGAP-IG, there were concerns in Nigeria about phone reliability, as well as reluctance by some staff, especially those nearing retirement, to adopt new ways of working.

Other LMICs studies on use of digital technology in healthcare [20–22] have tended to focus on health domains other than mental health. In an exploratory mixed method process evaluation sub-study to a cluster RCT on the impact on mobile phones on HIV/AIDS care in rural Uganda [21], qualitative data suggested broad support by frontline healthcare workers for the use of mobile phone applications to support their work. Similar to our findings, the Uganda study [21] also found phone reliability as a key barrier to mobile application use. Current use of mobile technology by frontline healthcare workers in Uganda [21] and other LMICs [20] was also for purposes other than clinical consultation and decision making.

We note that healthcare workers included in the present study had received training on the mhGAP-IG v1.0 and were expected to deploy the tool for identification and treatment of mental disorders in primary care. However, despite training and official expectations to use clinical guidelines for their work, the use of the mhGAP-IG v.1.0 and other existing paper guidelines were not embedded in current practice. A similar finding has been reported in Nepal [23] where despite an initial increase in detection rates for mental disorders after the introduction of the mhGAP-IG v.1.0, rates of detection reduced substantially two years later. This finding suggests that despite an initial excitement with the introduction of the mhGAP-IG v.1.0, use of the clinical guidance was not embedded in day-to-day practice. Due to previous observations of what is often referred to as ‘novelty effects’ of new interventions [20, 23], it is imperative to exercise caution about the enthusiasm expressed by primary healthcare workers in the present study around the perceived benefits and potential acceptability of a smartphone-based clinical guidance such as the mhGAP-IG v2.0. Some authors [20] have suggested the deployment of a user-centred design process to embed new interventions in primary and community care. In this process, frontline healthcare workers are continually engaged, feedback on their performance while at the same time taking note of their experience, contexts and constraints of use. As reported by participants in the present study, a somewhat similar process of engagement (i.e., calls from supervisors if not complying with directives to use designated apps) is currently in place in Nigerian primary care settings. As reported, this process has led to substantial compliance with official directives, and healthcare workers are getting used to the new way of working.

We identified other potential barriers to implementing smartphone-based clinical guidance and decision-making tools in Nigerian primary care settings. For example, we found that it was part of patients’ expectation for staff not to use their phones during consultation. Furthermore, and similar to existing paper guidance, time and work conflict in busy clinics may not afford the average primary healthcare worker sufficient time to consult a smartphone-based decision-making tool. Also, the currently embedded practice of paper recording of clinical consultation would require that healthcare workers duplicate records of the smartphone consultation in a paper format, thus adding to consultation time.

Time and work-related conflict as well as limited familiarity with smartphone use especially by older health care workers have often been reported in the global literature as key barriers to deployment of mobile technology. In a systematic review of published literature from Europe, North America and Asia, Berenguer and Colleagues (2017) found that in general, older people showed substantial barriers to smartphone adoption. According to the same study, those adopting smartphones often use it for calls and text messaging, and rarely did they use downloaded applications [24]. Studies conducted in India [25] and Nepal [25] also report poor staff familiarity with smartphones as a key barrier to the delivery of mental care by frontline healthcare workers using the platform. Apart from technology gaps, lack of the required infrastructure such as broadband and power supply, cost of running mobile phones, lack of policy support and patients concern about confidentiality and safety of their information ranked among the other top barriers to the deployment of digital technology for healthcare in LMICs [22].

Participants suggested important ways to scale the barriers to future use of app-based guidance including the emhGAP-IG in Nigerian primary care settings. For example, it was suggested that use of emhGAP-IG could be facilitated by training of staff, incorporating support and supervision, as well as explaining the reasons and process of its use before and during usual consultation. In keeping with primary care workers suggestion on the facilitatory effect of training, a systematic review on the feasibility and effectiveness of mobile health strategies for frontline health workers in LMICs [20] reported from 27 eligible studies that after adequate training of frontline healthcare workers, they were able to use mobile technology to provide a variety of healthcare interventions. An important finding in the present study is the report of participants suggesting that training of healthcare workers on the use of a future smartphone-based decision-making tool should be a reoccurring event.

Similar to findings in the present study, the prior Nepal study also reported that regular supervision from mental health specialists was an important facilitator of use of mobile technology by frontline healthcare workers [15]. A key difference reflecting contexts of supervision in the two countries is the aspiration of Nigerian PHCW for supervision by general practitioners, rather than by mental health specialists, to be more embedded in their practice. Incorporating support and supervision in a smartphone mhGAP-IG may be facilitatory in several ways. First, support and supervision of clinical activities are currently inadequate in Nigerian primary health settings. The current practice is for a supervisory general practitioner to provide unstructured support to a group of 6–8 primary health clinics in a local government area. However, actual supervisory sessions are rarely provided due to the time required by supervisory physicians to travel to conduct such sessions. Another way in which remote supervision may facilitate adoption of a smartphone mhGAP-IG is through healthcare workers cognizance of remote of oversight by a supervisor with mental health training who, as was reported in the present study, may be able to call the healthcare worker at any time may encourage use of the app. The main limitation of the present study is that it was conducted among staff in 12 PHCs in Southwestern Nigeria, and as such findings may not be generalisable to all Nigerian primary health care settings.

## Conclusion

We have found that the emhGAP-IG would be preferred to the paper format by PHCWs in our study settings. It would appear from our findings that emhGAP-IG could be a viable way to embed clinical guidance and decision-making tools for mental healthcare in the Nigerian primary health system. Future use of the e-mhGAP-IG would be facilitated by training and re-training of staff, helpful design features, and obtaining patients support by explaining the process, and the benefits during clinical contact. Embedding clinical guidance in primary healthcare may result in sustained improvement in detection rates and treatment of mental health conditions in LMICs.

## Supplementary Information

**Additional file 1: Appendix 1. **Draft interview topic guide forthe study.

## Data Availability

The datasets used and/or analysed during the current study are available from the corresponding author on reasonable request.

## Abbreviations

LMICs: Low- and Middle-Income Countries
RCT: Randomised Controlled Trials
MhGAP-IG: Mental Health Gap Action Programme-Intervention Guide
WHO: World Health Organization
GSMA: Groupe Speciale Mobile Association
SSA: Sub-Saharan Africa
EXPONATE: Expanding Care for Perinatal Women with Depression
MRC: Medical Research Council
EMILIA: E-mhGAP Intervention guide in Low and middle-income countries: proof-of-concept for Impact and Acceptability
PHCWs: Primary Health Care Workers
PHC: Primary health clinic
KII: Key Informant Interviews
FGD: Focus Group Discussions.
*LGA*: Local Government Areas
CHO: Community Health Officers
CHEW: Community Health Extension Worker
