# Supplementary material for: Perception of providers on use of the WHO mental health Gap Action Programme-Intervention Guide (mhGAP-IG) electronic version and smartphone-based clinical guidance in Nigerian primary care settings
Source: BMC Prim Care. 2022 Oct 15;23:264. doi: 10.1186/s12875-022-01869-7 (PMC9571457; doi:10.1186/s12875-022-01869-7)
Supplement: Supplementary file 1 — Additional file 1: Appendix 1. Draft interview topic guide forthe study. [file 12875_2022_1869_MOESM1_ESM.docx]

**Appendix 1: Draft interview topic guide for the study**

***Example introduction****: Hi, I’m SAY NAME and this is SAY NAME of note taker if appropriate]. Our team are talking to health care workers to find out ways we can develop technology to support you in your work in recognizing and supporting people with mental health issues.*

*To help us with this study, I would like to ask you questions to help the team understand more about your clinical situation, your use of technology and how technology may help or support you in your work.*

*Please feel free to ask me any questions during this interview and only express what you feel comfortable sharing with us. If there are any questions you are uncomfortable with, please let me know., You do not have to answer questions that makes you feel uneasy.*

*There are no right or wrong answers – our team are not here to test your knowledge in any way, we are interested in your personal opinions and experiences.*

**Demographics**

First of all, I’d like you to complete this demographic questionnaire, with a bit of information on your background as healthcare worker (GIVE INTERVIEWEE DEMOGRAPHIC QUESTIONS FOR COMPLETION.

The following provides an outline of the key topics to be covered in an interview.

| 1. **Warm up section:** *(5-10minutes): This is designed to learn some basic information about them and get them comfortable talking with you.* |
| --- |
| Review the completed demographic questions with the interviewee |

| 1. **Daily work life:**  *To find out about their regular day at work and how they assess someone with depression. 15 minutes.* |
| --- |
| 1. Can you describe to me your clinic setting and talk me through the patient journey, from arrival through to leaving the clinic? (prompts: focus on the reality observed in each setting. 2. Can you describe to me how people get referred to this clinic (in general)? How do people come seek care? 3. How many patients do you usually see in a day? 4. How many people did you see on your last typical work day (e.g. yesterday, or previous last work day).    - Is that normal? How many people do you normally see? 5. Tell me about a typical non-mental health consultation. What happens and how long do you normally spend with a patient 6. And how long do you spend if it is a mental health consultation?    - What are the main differences if you are doing a mental health or non mental health consultation? 7. Think back to the last patient you saw with depression. Please can you tell me about the clinical encounter. Please talk me through how they arrived, your assessment, how you identified depression etc.   Follow up questions   1. Describe the person (man, aged 40, married, referred by wife) 2. How did you start the consultation? 3. What did you talk about? 4. That’s interesting, how long did you spend talking 5. What did you talk about? 6. How did you diagnose depression? What information or guidance did you rely on? 7. What did you do afterwards?   Ask: was this a typical encounter? If not, how did it differ from a typical encounter. How did the depression presentation differ from other presentations? |

| 1. **Tech use section:**  *Understanding their technology use 10 mins* |
| --- |
| 1. What technology devices do you use regularly? (e.g. computer, smartphone, tablet, other)   For each device mentioned ask:   - - - - For what purposes (e.g. messaging, calling, writing, presentations etc)       - How often each day / week       - (if not covered): do you own or use a smartphone? What is it and how do you use it?   What social media or communication services you regularly use (e.g. what’s app, Facebook messenger, telephone etc).  For each communication service mentioned ask:   - - - - For what purposes (e.g. phone, text, voice message)       - For work or for leisure, connecting family etc.       - How often each day / week         - How many times in a day?         - How long do you talk for?   Additional specific questions to ask:  **Youtube:**   - - - - (If not mentioned, specifically ask):         - Do you use Youtube or other online video services?         - What do you use Youtube for?         - Do you ever use it for learning new things? If so, what was the last thing you learnt using it?   **Messaging:**   - - - - What messaging apps do you use?       - If / when you use messenger apps (e.g. whats app, viber etc), what do you use them for?         - How long do you use message for in a session, for example is it like a conversation or more just a brief message?         - How much text do you type? Do you ever write long messages using a smartphone?         - Do you ever have conversations using messenger apps?   **Data access:**   - - - - In terms of data use, how do you access the internet / social media?         - Do you use 3g / mobile data? If so, how do you use it.         - What do you use mobile data for?         - Are there any restrictions to its use?         - Do you use wi-fi at home, or wi fi elsewhere?         - Are there any restrictions to its use?         - Do you have data access at work? What do you use it for?   What other computer programs or software do you use regularly?  For each communication service mentioned ask:   - - - - For what purposes       - How often each day / week |

| 1. **Supervision /clinical mentoring:**  *To find out about their use of and interest in supervision 15 mins* |
| --- |
| In this section I would like to now talk about supervision. By supervision I am meaning clinical mentoring / clinical supervision (e.g. getting help with your work), but I will also ask a few questions about more management related supervision.   - 1. How often do you receive any type of formal supervision (e.g. meeting with a more experienced colleague, reporting back on work, cases seen etc).?   2. Can you describe to me how often this is and what you cover.   3. When did you last receive formal clinical supervision / mentoring for any type of health care? What happened during this?   4. And what about clinical supervision for mental health?   5. In the last year, approximately how many clinical supervisions / mentoring have you had? How long were they on average?   And how many on mental health  For clinical supervision / mentoring try to explore:   - - 1. What is the format/structure of your supervision?     2. How long does the session last for?     3. What kind of help do you receive?     4. What do you learn during the supervision? (prompt, e.g. clinical, professional development, support, problem solving etc)   Now I’d like you to think of less formal ways you learn and get support in your work:   1. How would you go about asking for help with a clinical situation that is challenging/? difficult. For example, if you needed help with a clinical case, what would you do. 2. Can you describe to me please the last time you felt you needed help from a supervisor or more experienced colleague with a patient who lived with a mental illness? 3. What do you consider to be a challenging case. Can you give me an example of a challenign case with depression? What features made it challenging?    1. What would you consider a difficult case, when would you seek supervision or support for? For what problems?    2. (if not already covered) - What about informal clinical mentoring or support, for example speaking to a colleague, information sharing at a team meeting. How often is this and what happens    3. Do you ever speak to a colleague or someone on the phone for support? When was the last time you did this and what happened. 4. If you want to learn something new, such as a new clinical procedure or new way of working, how would you learn this?    - 1. Do you ever self-learn? If so, how do you do this?      2. Do you ever use e-learning, online videos, or anything else?         1. When did you last do this and for what? |

| 1. **Identifying key problems experienced at work** *Aim of activity: to identify the main problems that the participant faces and to sort in order of preference what s/he would (i) like most support with, and (ii) how s/he would like to access that information.* |
| --- |
| - For this exercise, you can ask the participant to list all the problems they face, or you can write them on cards for later sorting. - Ask participant to list all the problems they commonly face in their work - Once a list is made, ask them to rank in order of preference HCW would like help and support with. Rank all the problems in order of priority, starting with the most important / challenging problem at the top of the list - If participants have difficult coming up with ideas, you can prompt:   - Difficulty in identifying depression or other mental health conditions   - Ensure I identify r all the symptoms necessary to make a diagnosis   - Remember the sequence/logic steps to disease management   - Time management when experience a heavy workload   - Remembering what. Happened in the last session - For each of the ranked problems ask:   - How often does this happen   - When did? It. Last happen to you?   - Was this more or less of a problem at any other time of your work, for example when you were new to the work, or when you changed role of had more responsibility?   - What was the outcome - How would your work life change if you were to address this problem? - Do you have any ideas on how you could address this problem?   - How could this problem be addressed by your manager? / supervisor?   - Is there any way technology could be used to address this problem? - if there are no problems that will be addressed through EMILIA in the top 5 (e.g. making an accurate diagnosis, follow ups, support and supervision) ensure you also cover these.   *Introduction*  We will now talk about common problems you face in your work. There are no right or wrong answers, and you can be as specific or general as you like. Once we’ve listed them all I will ask you to order them by priority, starting with the most important / challenging problem at the top of the list. Is that clear? Do you have any questions?  Prioritise top top 5 – now please choose the top 5 that have the biggest negative impact on your service / top 3 or 5 that we could change that would be most helpful for you and can be under our control. |

| 1. **Use of technology at work:** *Aim of questions: obtain an idea of how mhGAP can be adapted* |
| --- |
| I’d like to ask you a bit more about the use of apps and record keeping at work. This is to help us design an electronic version of mhGAP for use by HCWs.  (if not already covered elsewhere)   - Can you tell me a bit about how you keep records. For example, do you use an OPD register, or keep case files? Please describe these to me.   - What information do you record and where?   - How is this information used (e.g. for follow up).   - How do you keep information of follow up appointments?  1. Can you tell me what your views are about these following issues:    1. Do you use the mhGAP book (or any other book) during a consultation to help you make a diagnosis?       1. Please explain why / why not:    2. Would you use a smartphone app during a consultation to help you make a diagnosis?       1. Please explain why / why not:    3. Do you write clinical notes during a consultation when with a patient?       1. Please explain why / why not:   I’d like to ask you about any Apps you use at work:   - - - - Have you ever used an app at work, for example for treating a patient (examples may include, apps for checking drug dosage, IMCI, diagnosis support tools etc).       - What was the app, what was its name and what does it do?       - Did you use this while a patient was present?       - How often do you use these apps?       - When did you last use it? - We are planning to update an electronic version of mhGAP that will help HCWs in identifying, assessment and managing mental health conditions. At first, we will focus on depression. - First of all, what do you think of this idea (e.g. do they think it is a good idea or not?) - What features or designs would you like to see?   - If person has difficulty answering this you could say:     - For example, you know the mhGAP 2.0 book. How can we make this into something that would help HCWs, can you describe this to me?     - e.g. would it help you identify depression; how would it do this?     - Would it help you with supervision or to get expert help, how would it do this?     - What features would help you in your work or make your life easier? - Would it be feasible for health workers working in government health centres to use this? (feasible and acceptable) |
